# Development and validation of the cancer symptoms discrimination scale: a cross-sectional survey of students in Yunnan, China

**DOI:** 10.1186/s12904-020-00662-6

**Published:** 2020-10-12

**Authors:** Lin-sen Feng, Zheng-jiao Dong, Ruo-yu Yan, Chang-ling Tu, Lan-yu Zhang, Jiang-yun Shen, Shi-yu Zhang

**Affiliations:** 1 The Sixth Affiliated Hospital of Kunming Medical University (The People’s Hospital of Yuxi City), Yuxi, Yunnan China; 2 School of Public Health, Kunming Medical University, Kunming, Yunnan China; 3 The Third Affiliated Hospital of Kunming Medical University (Yunnan Cancer Hospital), Kunming, Yunnan China; 4 No.1 School of Clinical Medicine, Kunming Medical University, Kunming, Yunnan China

**Keywords:** Cancer, Symptoms discrimination, Scale, Influencing factors, Cross-sectional survey

## Abstract

**Background:**

This study aimed to devise a Cancer symptoms Discrimination Scale (CSDS) suitable for China based on a cross-sectional survey.

**Methods:**

The CSDS was developed using the classical measurement theory. A total of 3610 students from Yunnan province, China, participated in the cross-sectional survey. The test version of the scale was modified by the item analysis method, and after the official version of CSDS was developed, its reliability and validity were verified. A univariate analysis of variance and a multiple linear regression model were used to analyze the influencing factors of cancer symptoms discrimination among the university/college students.

**Results:**

There were 21 items in total for the CSDS, including 3 subscales --- common clinical manifestations (11 items), physical appearance defects (6 items), and drainage tube(s) wearing (4 items). This CSDS had good validity (GFI = 0.930, AGFI = 0.905, RMR = 0.013, I-CVIs> 0.80, and the Pearson correlation coefficient was satisfactory.) and reliability (Cronbach’s alpha = 0.862, spearman-brown coefficient = 0.875). The multiple linear regression showed that certain factors may affect the students’ discrimination level against cancer symptoms (*P* < 0.05), including gender, major, current education degree, guardian’s highest record of formal schooling, self-rated health status, history of care for cancer patients, family relationship, ways of cancer knowledge acquisition, good/poor understanding of cancer-related information, degree of cancer fear, and their perception of cancer infectiousness.

**Conclusion:**

This CSDS, with good reliability and validity, can be used for the evaluation of the discrimination risk and levels against cancer symptoms among healthy students.

## Background

The national cancer monitoring report released by the national cancer center of China at the beginning of 2019 says that among every 100,000 Chinese, 285.83 people suffer from cancer, and the death rate is 170.05 per 100,000 [1]. Therefore, it has becoming a necessity to enforce the health management of the whole life cycle in cancer patients, so that the improvement of the survival rate can be achieved. The health management of cancer patients should be patient-centered. A comprehensive multidisciplinary mode, including clinical, psychological, and social sciences, makes it possible to provide an all-round service for the patients.

Cancer has a high incidence, low cure rate and high mortality. Cancer often causes serious threats to patients’ mental and physical health [2–4], diminishes their quality of life [5, 6], and social support for the patients decreases along with the progress of the disease [7–9]. Though it causes heavy disease burden [10], it arouses low public awareness [11, 12]. In today’s society, the public tend to avoid disadvantages while seeking advantages both in their ideological and behavioral activities. Therefore, with strong self-protection instincts, healthy people may generate isolation, rejection, belittling, stigmatization and other negative attitudes and behaviors against cancer patients [13–15]. It is a fact that cancer patients, as a special and vulnerable group related to major diseases, may suffer cancer discrimination from the public. An epidemiological survey showed that southwest China is a “hard-hit area” of cancer in China, ranking first among the seven geographical regions in terms of cancer incidence and mortality. The incidence and mortality among rural residents are higher than those in urban residents [16]. For example, Xuanwei, Fuyuan [17, 18] and Gejiu [19] in Yunnan province are regions with high incidence of lung cancer recognized by medical communities in both Chinese and other countries. And above reasons may further aggravate the public’s biased perception of cancer, and even lead to the stigmatization and marginalization of cancer patients.

Discrimination has various forms, including social system structural discrimination, gender discrimination, racial discrimination and so on. Disease discrimination is a kind of widespread and universal discrimination, but it is usually ignored. A large number of previous studies has confirmed that the main “victims” of disease discrimination include the pathogen carriers and patients of chronic infectious diseases such as tuberculosis [20], AIDS [21] and hepatitis B [22], as well as patients with mental diseases such as depression and schizophrenia [23, 24]. Discrimination might be found in general social groups as well as health care professionals [25, 26]. Unlike mental disease and infectious disease discrimination which have gained much attention, only a very little research focused on cancer discrimination both in China and other countries.

Cancer discrimination refers to the negative, differentiated, marginalized, unfair and exclusive ways that cancer patients are treated by healthy people because of their disease [27]. There is a sense of acceptance in healthy people, but healthy people may hold social, cultural and value discrimination against a small number of people who have cancer. Healthy people may demonstrate their cancer discrimination in their attitudes and/or behaviors. For example, healthy people who were informed that cervical cancer may be related to HPV were more likely to consider cervical cancer patients as dirty, thoughtless and dishonest, and they would hold discrimination and blame against the patients [28]. In addition, since some pathological lung cancer has been proved to be closely related to smoking, people in some western countries tend to project the prejudice and discrimination against the smokers on lung cancer patients, thus causing a severe sense of shame in lung cancer patients with a history of smoking [29]. It is safe to say that people are very likely to transfer their moral aversion and stigma, such as “sexually transmitted diseases, second-hand smoke hazards”, to cancer patients, thus breeding discrimination against cancer patients when people only partially understand the relationship among factors such as HPV infection, smoking, and cancer. Hence, cancer discrimination may stem from healthy people’s moral aversion and blaming attitudes toward certain cancer triggers, such as unclean sex and smoking. Also, fear of death may be another factor resulting in cancer discrimination [30].

Previous studies have found that the adverse effects of cancer symptoms/side effects in cancer patients mainly include worries about the future, emotional difficulties, decreased physical functions, impaired sexual function, negative self-image, fatigue, sleeping difficulty, financial burden and work limitations [31, 32]. According to our own previous research, we hold the opinion that one main cause of cancer discrimination is the discrimination against cancer symptoms in healthy people [33, 34]. The easy-to-be-noticed cancer symptoms are the primary differences between cancer patients and the healthy people [35, 36]. Healthy people may label cancer patients as “dangerous”, “marginal” and “different” because of their fear, rejection and avoidance of cancer symptoms. Eventually cancer patients become victims of discrimination related to cancer symptoms. This is the definition of cancer symptoms discrimination. Therefore, an effective scale suitable for the public is urgently needed, so that it could be possible to evaluate the levels of discrimination against cancer symptoms among the general public in China, and to propose relevant intervention strategies for cancer symptoms discrimination in China.

This study aimed to devise a Cancer symptoms Discrimination Scale (CSDS) suitable for China based on the empirical investigation and research. The healthy medical and non-medical university/college students were investigated by a questionnaire. The reliability and validity of the CSDS were verified. A system of cancer discrimination risk evaluation was initially established. This CSDS was preliminarily used to measure the degree of discrimination against cancer symptoms among university/college students, whose influencing factors were also analyzed. It could provide theoretical basis for future intervention.

## Methods

### Development of the pilot version of the CSDS

Our research group devised the CSDS with classical measurement theory. (1) Semi-structured interview: Being given the topic that “your views on cancer symptoms and cancer patients”, 26 college students were invited to have a one-to-one, direct and in-depth semi-structured interview (see Additional File 1). The sample size of the semi-structured interview was determined by “information saturation method”, that is, the sample size of participants was determined when no further information could be obtained. In the semi-structured interview, the interview was terminated once no more new and effective information could be obtained from more participants [37]. (2) Literature reviewing: Retrospective analyses of literature and mature scales related to AIDS, mental disease and other disease discrimination were made, in order to learn their experience in scale developing. (3) Delphi method: We invited 3 oncology experts and 2 nursing experts for the expert consultation. With a combination of the above methods, the item pool was formed by fully collecting the effective information, and the pilot scale was developed.

The CSDS beta contained 21 items, each reflecting the participants’ subjective-perceived degree of avoidance, rejection and unwillingness to approach against some common signs and symptoms of cancer patients. In this way, the risk of discrimination against the common symptoms and clinical manifestations of cancer was measured. Summated rating scale scoring was adopted. All items were scored in the same direction, i.e. “1 = yes” and “0 = no”. The higher the score, the greater the risk of discrimination was.

Cognitive interviewing was used to assess the suitability of all items for the formal investigation by inviting 15 participants each round for the “verbal probing”. In the first round, 15 participants completed each item in the scale and answered the question “Can you repeat what you just read in your own language?”. Needed modification of individual words was made according to the interviewee’s feedback. After that, another 15 participants conducted “verbal probing” again to ensure that each item was concise, clear, accurate and unambiguous. Further modification would be made if any ambiguous word existed according to further feedback. Then another 15 participants would be invited for the next round of cognitive interviewing.

### Selection of participants

The participants’ inclusion criteria included: Students who were (1) full-time students in universities/colleges or secondary vocational schools; (2) currently freshmen, sophomores or juniors; (3) voluntary participants in this survey.

The exclusion criteria were: Students who were (1) reluctant to participate in the survey; (2) victims of major family changes in half a year; (3) victims with a history of malignant tumors.

### Data collection

The questionnaire consisted of two parts: (1) A questionnaire for the demographic and sociological characteristics, including basic information such as gender, age, school, major, grade, residence, number of family members, monthly family income, guardian’s highest record of formal schooling, occupation, self-rated health status, ways of cancer knowledge acquisition and so on; (2) The CSDS.

Five schools in Yunnan province were taken as the field investigation sites. Among them, school A is a general university of medicine and health, school B is a comprehensive university of higher learning, school C is a vocational college of medicine and health, school D is a comprehensive secondary vocational school, and school E is a secondary vocational school of medicine and health. Based on the school’s total students’ population, and the inclusion criteria developed by the research group, the number of students from each school was determined using the quota sampling method. Students who met inclusion criteria were chosen as the study subjects. The investigators included members of the research group, some school teaching administrators and in-service teachers, who had passed an organized training and were qualified. Before issuing the questionnaire, the students were informed of the purpose and significance of the survey with the same guideline. The principles of voluntary, anonymity, confidentiality and irrelevance to cancer treatment were made clear to the students. After giving their informed consents, the students completed the questionnaire by themselves. The questionnaires were collected by the investigators on location.

### Item analysis

To further improve the scale, the following two methods were used to eliminate the items of poor quality. (1) Correlation analysis: The score of each item in the pilot version and the total score were analyzed by Person correlation, and the items with correlation coefficient γ < 0.3 or *P* < 0.05 were deleted. (2) Cronbach coefficient method: After deleting each item, the change of Cronbach coefficient α was observed. The items that significantly reduced the α value of the scale were deleted. The remaining items were then renumbered to form a formal scale.

### Reliability and validity test of the CSDS

The reliability and validity of the official CSDS were tested. A structural equation model was constructed and the scale’s structural validity was tested by confirmatory factor analysis. When it comes to the test of fitting degree in large sample studies, the chi-square value is often very big resulting in a very small α test level, causing rejection of models actually with good fitting degrees. With the sample size *N* ≥ 1000 in this study, the chi-square criterion was not used in the fitting degree test. GFI, AGFI, RMR, RMSEA, CFI, NFI, TLI, RFI, PNFI and PGFI were used as the fitting degree test indicators [38]. The composite reliability (CR) value of each factor was calculated to evaluate the convergent validity of this CSDS. Pearson correlation analysis and expert consultation for I-CVI values were used to evaluate the content validity of the scale.

Cronbach α was used to evaluate the internal consistency reliability of the scale. Spearman-brown Coefficient and Guttman Split-Half Coefficient were used to evaluate the semi-reliability of the scale.

### Analysis of influencing factors of cancer symptoms discrimination

Descriptive analysis was used to analyze the discriminatory attitudes towards cancer symptoms among university/college and secondary vocational school students.

Univariate analysis of variance and multiple linear regression analysis were used to analyze the influencing factors of the CSDS score, and to screen the risk factors of cancer discrimination among the university/college and secondary vocational school students. A multiple linear regression equation model of influencing factors of cancer discrimination among the university/college and secondary vocational school students was constructed.

The cancer symptoms discrimination score (Y) was used as the dependent variable. And the independent variables (X) included gender, age, nationality, school, major, grade, current education degree, family residence, family income, number of family members, et al. The independent variable whose *P* < 0.1 was put into the regression equation. Stepwise regression was used for analysis, with the entry probability α being 0.05 and the exclusion probability α being 0.10.

SPSS Statistics 19.0 and SPSS AMOS 22.0 were used to analyze the collected data. The test level was α = 0.05, and all *P* values represented bilateral probability.

## Results

### The devising of the CSDS pilot version

After the semi-structured interviews and literature reviewing, the research group screened out 21 cancer signs and symptoms that might cause discomfort, disgust, avoidance and rejection in healthy people. We put them in plain language, and devised the CSDS pilot version. It was divided into three subscales: common clinical manifestations, physical appearance defects and drainage tube(s) wearing. The wording of the items didn’t change after the first round of expert consultation. In the second round of expert consultation, the I-CVI values of all 21 items were greater than 0.80. Then, the participants did not put forward any suggestions on the revision of the CSDS pilot version after two rounds of cognitive interviews.

### Sample size

A total of 3800 questionnaires were distributed at 5 schools (1450 in school A, 800 in school B, 800 in school C, 320 in school D, and 430 in school E), and 3723 copies were collected, among which 3610 were valid (1411 in school A, 753 in school B, 750 in school C, 299 in school D, and 397 in school E), with an effective rate of 95.0%. The median age of the participants was 19 (range from 16 to 29). The whole questionnaire was completed in 4–8 min.

### Item analyses

Pearson correlation analysis indicated that the correlation coefficient r between the scores of the 21 items and the total score of the CSDS pilot version were all > 0.30 and with *P* < 0.05. r ranged from 0.414 to 0.610 with *P* < 0.05. No item could be deleted in this method. Cronbach’s α in the pilot version = 0.862, and the deletion of any item would cause the reduction of the Cronbach coefficient α of the scale, so no item could be deleted in this method.

In combination with the above two methods, all 21 items in the CSDS pilot version were verified, and the official CSDS version with 21 items was developed.

### Validity test

#### Structural validity and convergent validity test

**Fig. 1:**
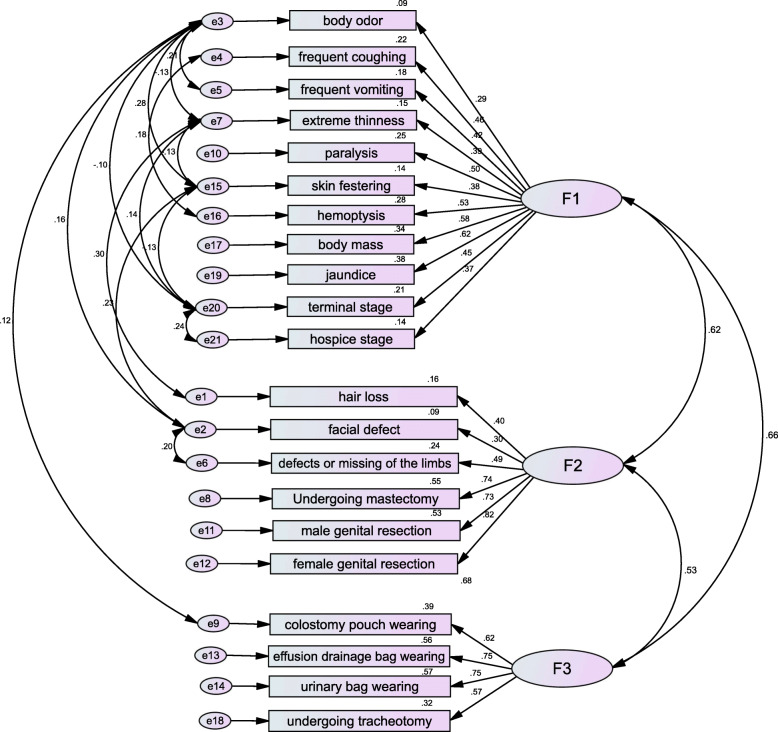
The corrected CSDS three-factor structural equation model (F1: common clinical manifestations. F2: physical appearance defects. F3: drainage tubes wearing)

The result of KMO and Bartlett test of sphericity (KMO = 0.899, Bartlett significance *P* < 0.001) indicated perfect appropriateness to further conduct the confirmatory factor analysis [39]. The structural equation model showed that the CSDS three-factor model was consistent with the designed dimensions, and the fitting degree of the corrected three-factor model was better than the uncorrected three-factor model, suggesting that the scale had good structural validity (see Table 1, Fig. 1). The first factor (F1) was named “common clinical manifestations”, which mainly described the discriminatory attitudes and behaviors of healthy people towards various common clinical manifestations of cancer, such as avoidance, refection, isolation and aversion. The second factor (F2) was named “physical appearance defects”, which mainly described the discriminatory attitudes and behaviors of healthy people towards the physical appearance defects caused by cancer treatment in cancer patients. The third factor (F3) was named “drainage tube(s) wearing”, which mainly described the discriminatory attitudes and behaviors of healthy people towards cancer patients who wore colostomy tubes (bags), serous cavity drainage tubes, urinary tubes or trachea cannula.

**Table 1:** Fitting degrees of the CSDS structural equation model

	GFI	AGFI	RMR	RMSEA	CFI	NFI	TLI	RFI	PNFI	PGFI
**Uncorrected three-factor model**	0.864	0.832	0.016	0.082	0.782	0.776	0.754	0.747	0.687	0.696
**Corrected three-factor model**	0.93	0.905	0.013	0.064	0.88	0.858	0.873	0.845	0.715	0.692
**Threshold**	>0.90	>0.90	<0.05	<0.08	>0.90	>0.85	>0.85	>0.85	>0.50	>0.50

The convergent validity test results showed that the composite reliability value of F1 was 0.744, F2 was 0.765, and F3 was 0.771, all of which were greater than 0.7, indicating that each factor of this CSDS had good convergent validity.

### Content validity test

The I-CVI values of all 21 items of the CSDS were greater than 0.80 after expert consultation. The results of Pearson correlation analysis on the scores of each item, the score of the subscales and the score of the total scale indicated that each item had a good correlation with the total scale, ranging from 0.435–0.610. The correlations between each subscale and the total scale were excellent, ranging from 0.745 to 0.915. The correlations between each item and its subscale were better than those between other subscales. The above results all suggested that this CSDS had a good content validity (see Table 2).

**Table 2:** Correlation analyses of the CSDS total scale, subscales and each item

	CSDS	Common clinical manifestations	Physical appearance defects	Drainage tubes wearing
**Subscales**				
**Common clinical manifestations**	**0.915**			
**Physical appearance defects**	**0.802**	0.591		
**Drainage tubes wearing**	**0.745**	0.520	0.481	
**Items**				
**Hair loss**	**0.478**		**0.598**	
**Facial defect**	**0.514**		**0.559**	
**Body odor**	**0.414**	**0.464**		
**Frequent coughing**	**0.490**	**0.586**		
**Frequent vomiting**	**0.492**	**0.565**		
**Defects or missing of the limbs**	**0.579**		**0.651**	
**Extreme thinness**	**0.435**	**0.475**		
**Undergoing mastectomy**	**0.517**		**0.734**	
**Colostomy pouch wearing**	**0.537**			**0.765**
**Paralysis**	**0.524**	**0.519**		
**Male genital resection**	**0.523**		**0.690**	
**Female genital resection**	**0.550**		**0.747**	
**Effusion drainage bag wearing**	**0.594**			**0.788**
**Urinary bag wearing**	**0.588**			**0.807**
**Skin festering**	**0.463**	**0.520**		
**Hemoptysis**	**0.553**	**0.631**		
**Body mass**	**0.581**	**0.606**		
**Undergoing tracheotomy**	**0.565**			**0.704**
**Jaundice**	**0.610**	**0.619**		
**Terminal stage**	**0.476**	**0.508**		
**Hospice stage**	**0.435**	**0.470**		

### Reliability test

The results suggested a good reliability of this CSDS, with the Cronbach’s α of the total scale =0.862, and the Cronbach’s α of the three subscales (common clinical manifestations, physical appearance defects and drainage tube(s) wearing) = 0.760, 0.721 and 0.763, respectively. The coefficients of Spearman-brown and Guttman Split-Half of this CSDS were 0.875 and 0.873 respectively. The Spearman-brown coefficients of the three subscales (common clinical manifestations, physical appearance defects and drainage tube wearing) were 0.800, 0.766 and 0.764 respectively; and Guttman Split-Half of the three subscales were 0.791, 0.765 and 0.762 respectively (see Table 3).

**Table 3:** The CSDS Reliability Test Results

	Cronbach α	Split-Half reliability	
		Spearman-Brown coefficient	Guttman Split-Half coefficient
**CSDS**	0.862	0.875	0.873
**Common clinical manifestations**	0.760	0.800	0.791
**Physical appearance defects**	0.721	0.766	0.765
**Drainage tubes wearing**	0.763	0.764	0.762

### Descriptive analysis of discriminatory attitudes towards cancer symptoms

**Fig. 2:**
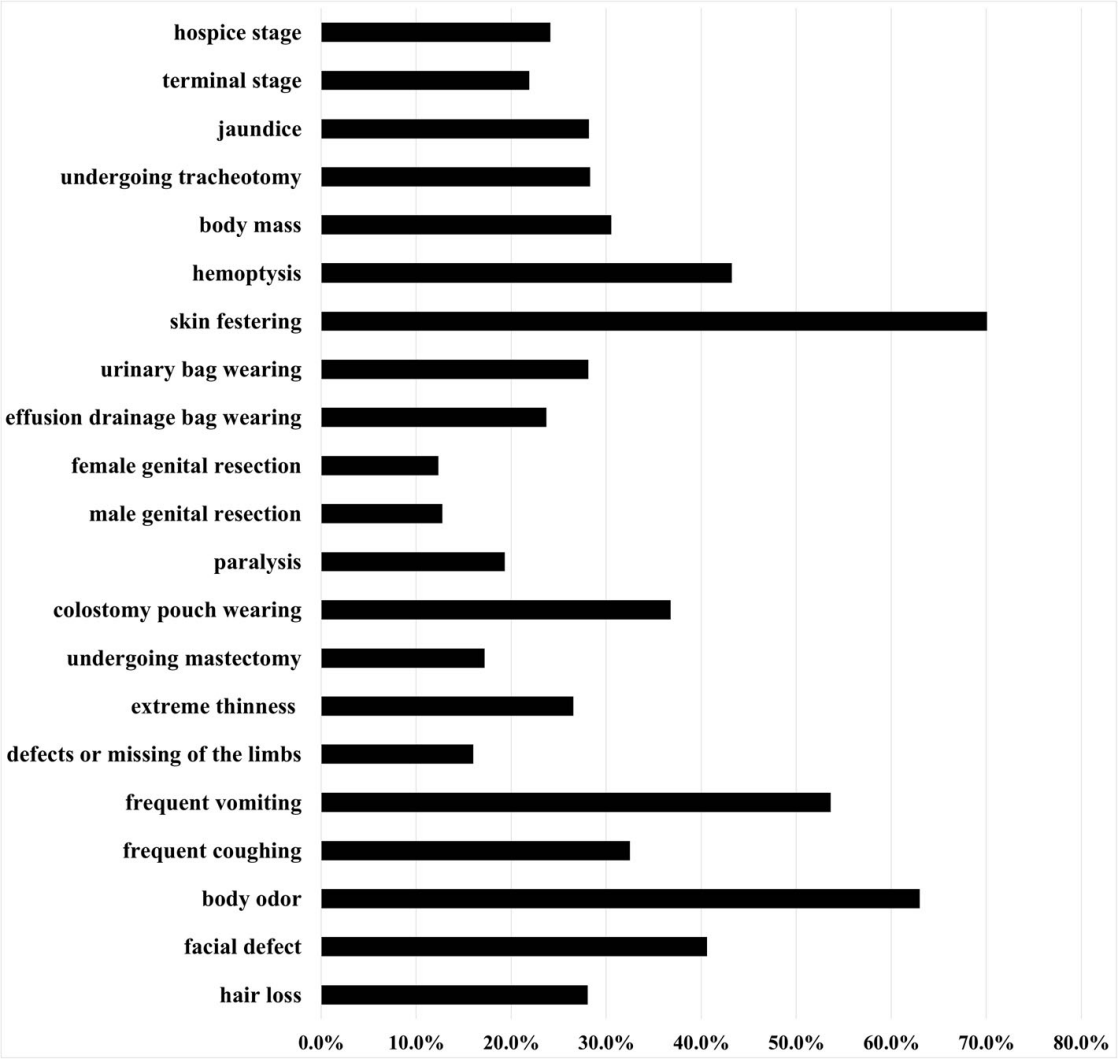
Percentages of the discriminatory attitudes and behaviors towards cancer symptoms among the university/college and secondary vocational school students (%)

The descriptive analysis showed that the top five symptoms causing discriminatory attitudes and behaviors towards cancer symptoms were skin festering (70.1%), body odor (63.0%), frequent vomiting (53.6%), hemoptysis (43.2%), and facial defects (40.6%). (see Fig. 2.)

### The univariate variance analysis of the influencing factors of cancer discrimination

We used a univariate analysis of variance to compare the effects of different demographic and sociological characteristics on the scores of the CSDS. Results found that the CSDS discrimination scores in different groups had statistically significant differences in terms of the participants’ age, school, major, grade, current education degree, family residence, number of family members, guardian’s highest record of formal schooling, self-rated health status, history of cancer in close relatives, history of care for cancer patients, family relationship, ways of cancer knowledge acquisition, understanding of acquired cancer knowledge, self-rated degree of danger from cancer, degree of fear of cancer, perception of whether cancer is contagious and other factors (*P* < 0.05) (see Table 4).

**Table 4:** Comparisons of the CSDS scores of participants with different characteristics (Mean ± SD)

General characteristics		N	The CSDS score	***P***
**Gender**	**male**	917	6.81 ± 5.23	0.077
	**female**	2690	6.49 ± 4.57	
**Age**	**≤18 years**	1566	6.82 ± 4.81	0.008
	**>18 years**	2023	6.39 ± 4.68	
**Nationality**	**Han**	2710	6.48 ± 4.70	0.061
	**other minorities**	899	6.83 ± 4.88	
**School**	**School A**	1411	6.16 ± 4.78	<0.001
	**School B**	753	6.15 ± 4.35	
	**School C**	750	6.50 ± 4.46	
	**School D**	299	7.34 ± 4.78	
	**School E**	397	8.38 ± 5.32	
**Major**	**clinic medicine**	1182	5.85 ± 4.43	<0.001
	**nursing**	1017	6.92 ± 4.77	
	**other medical majors**	262	6.88 ± 5.28	
	**non-medical majors**	1149	6.92 ± 4.82	
**Grade**	**freshman**	1380	6.33 ± 4.48	<0.001
	**sophomore**	1438	6.99 ± 5.01	
	**junior**	791	6.21 ± 4.65	
**Current schooling degree**	**secondary vocational school**	668	7.88 ± 5.14	<0.001
	**specialized vocational college**	879	6.75 ± 4.67	
	**university/college**	2054	6.07 ± 4.55	
**Residence**	**rural area**	2337	6.78 ± 4.72	<0.001
	**urban area**	1250	6.16 ± 4.72	
**Family income**	**≤ 3500 yuan**	1638	6.68 ± 4.73	0.623
	**>3500 yuan**	1613	6.60 ± 4.78	
**Number of family members**	**≤3 people**	820	6.03 ± 4.73	<0.001
	**≥4 people**	2764	6.73 ± 4.74	
**Guardian’s education**	**Primary school and below**	745	6.86 ± 4.85	<0.001
	**middle school**	1656	6.80 ± 4.71	
	**high school/ secondary vocational school**	698	6.37 ± 4.78	
	**specialized vocational college**	232	5.55 ± 4.37	
	**university/college and above**	251	5.67 ± 4.66	
**Self-rated health**	**well**	1975	6.25 ± 4.66	<0.001
	**not well**	1627	6.97 ± 4.82	
**Having chronic diseases or NOT**	**no**	3392	6.56 ± 4.71	0.427
	**yes**	208	6.83 ± 5.28	
**History of cancer in close relatives**	**no**	3208	6.63 ± 4.77	0.027
	**yes**	399	6.08 ± 4.56	
**History of cancer in close friends**	**no**	3426	6.57 ± 4.73	0.926
	**yes**	182	6.54 ± 5.04	
**History of care for cancer patients**	**no**	3367	6.64 ± 4.75	0.001
	**yes**	231	5.59 ± 4.52	
**Family relation**	**harmonious**	3062	6.47 ± 4.67	0.002
	**inharmonious**	546	7.15 ± 5.12	
**Personality**	**introversive**	590	6.56 ± 4.66	0.957
	**non-introversive**	3011	6.57 ± 4.77	
**Ways of cancer knowledge acquisition**	**≤3**	1797	5.94 ± 4.34	<0.001
	**≥4**	1809	7.19 ± 5.03	
**Understanding of cancer knowledge**	**very**	658	5.77 ± 4.75	<0.001
	**fair**	2317	6.63 ± 4.67	
	**little**	628	7.19 ± 4.93	
**SELF-perceived cancer danger**	**so so**	1411	6.23 ± 4.44	0.001
	**very much**	2196	6.79 ± 4.92	
**Degree of cancer fear**	**very much**	2036	7.10 ± 4.88	<0.001
	**not so much**	1569	5.88 ± 4.47	
**Perception of cancer contagiousness**	**not contagious**	1992	6.22 ± 4.58	<0.001
	**not sure**	958	6.99 ± 4.94	
	**contagious**	654	7.02 ± 4.85	

### The multiple linear regression analysis of the influencing factors of cancer symptoms discrimination

A multiple linear regression analysis was used to further evaluate the effects of the above factors on the scores of the CSDS. The results showed that the multiple linear regression equation established with Y as the dependent variable was statistically significant (*F* = 26.761, *P* < 0.001). The collinearity diagnosis showed that the tolerance and variance expansion factor of 11 independent variables were close to 1, indicating that there was no collinearity problem among the independent variables.

Factors that may significantly affect the students’ level of discrimination against cancer symptoms were gender, major, current education degree, guardian’s highest record of formal schooling, self-rated health status, history of care for cancer patients, family relationship, ways of cancer knowledge acquisition, understanding of acquired cancer knowledge, degree of cancer fear, and perception of whether cancer is contagious (*P* < 0.05) (see Table 5). The model fitting test results showed that *R* = 0.280, *R*^2^ = 0.078. This low model fitting degree suggested that there would be other factors influencing the level of cancer symptoms discrimination, in addition to the independent variables included in this study.

**Table 5:** Multiple linear regression analyses of the CSDS scores

Variables	Regression coefficient	Standard error	Standardized regression coefficient	t	***P***
**Constant term**	6.345	0.823	—	7.709	<0.001
**Gender**	−0.552	0.180	−0.051	−3.065	0.002
**Major**	0.289	0.064	0.075	4.537	<0.001
**Current education degree**	−0.598	0.102	−0.099	−5.859	<0.001
**Guardian’s highest record of education**	−0.211	0.073	−0.048	−2.880	0.004
**Self-rated health**	0.568	0.160	0.060	3.551	<0.001
**History of care for cancer patients**	−0.877	0.317	−0.045	−2.769	0.006
**Family relationship**	0.510	0.220	0.039	2.317	0.021
**Ways of cancer knowledge acquisition**	1.267	0.156	0.134	8.110	<0.001
**Understanding depth of acquired cancer knowledge**	0.528	0.134	0.067	3.948	<0.001
**Degree of cancer fear**	−1.123	0.158	−0.118	−7.121	<0.001
**Perception of whether cancer is contagious**	0.371	0.101	0.061	3.683	<0.001

## Discussion

Based on the interview results, expert consultation and clinical experience, this research group developed a CSDS which could be used to evaluate the manifestation and severity of the discrimination against cancer symptoms in healthy people. A total of 21 items in the CSDS were divided into three subscales: common clinical manifestations (11 items), physical appearance defects (6 items), and drainage tube(s) wearing (4 items). The Cronbach coefficient α and the Split-Half coefficient of the CSDS were all > 0.85, the Cronbach coefficient α and the Split-Half coefficients of the three subscales were all > 0.70, suggesting good reliability and strong reliability in the total scale and the subscales [40, 41]. The confirmatory factor analysis showed that the corrected three-factor model had a better fitting degree compared with the uncorrected three-factor model, showing that the CSDS had a good structural validity. Increasing the correlation between some latent variables helps to improve the fitting degree. This suggested public’s discrimination against different cancer symptoms interacts with each other. In addition, the composite reliability values of the three factors were all > 0.70, and each factor was used to measure the same underlying trait, suggesting that each factor have good convergent validity. The I-CVI value was > 0.80 and Pearson correlation coefficient was at a satisfactory level, indicating that this CSDS had good content validity. This study took students from some universities/colleges and secondary vocational schools in Yunnan province as the research subjects and we conducted a cross-sectional survey. The samples are representative because most of the students are from different cities and counties under the jurisdiction of Yunnan province. The results of this study indicate that the public discrimination against the symptoms of cancer patients does exist. Descriptive statistical results suggested that there might be differences in the severity of discrimination in different student groups. Among the 21 cancer symptoms, skin festering (70.1%), body odor (63.0%), frequent vomiting (53.6%), hemoptysis (43.2%), and facial defects (40.6%) are more likely to encounter discriminatory attitudes and behaviors such as avoidance, rejection, and unwillingness to approach. The above five symptoms are more visible, and easier to be noticed, therefore are likely to arouse undesirable feelings such as “dirty”, “disgusting”, “fearful” and “unacceptable” than the rest symptoms. They are more likely to cause psychological impact to healthy people, more likely to be associated with “death”, hence are more likely to cause avoidant and repulsive attitudes or behaviors in healthy people. Relatively, female genital mutilation (12.3%), male genital mutilation (12.7%), limb defects (16.0%), and mastectomies (17.2%) may be less susceptible to public discrimination. In our opinion, that’s because the absence of a sex organ or a breast is a very private matter and could not be easily detected. There are many factors leading to limb defects (such as accidental injury and congenital defect), and some patients can improve their life quality by wearing artificial limbs and constant exercise of the residual limbs [42]. Then, public acceptance of such symptoms would be reasonably higher, and the associated level of public discrimination may be lower.

This CSDS scale was used to preliminarily evaluate the discrimination levels and influencing factors of the university/college and secondary vocational school students towards cancer patients. The univariate analysis of variance and multiple linear regression analysis found that students who were more likely to hold more cancer symptoms discrimination were: males, non-clinical medicine majors, with low current education degrees, with low guardian’s schooling degrees, with poor self-rated health, having no experience in care for cancer patients, having no harmonious family relations, having various access to cancer knowledge, with poor understanding of cancer, having extreme fear of cancer, considering cancer as contagious and not sure whether cancer is contagious. Taking the above results in consideration, we can conclude that the important inducements of cancer symptoms discrimination in college students are: poor family environment (including poorly educated guardians and dysfunctional family relationships) and lack of medical knowledge or relevant educational experience (including non-medical education background, low level of education, self-perceived poor health, lack of cancer nursing experience, poor understanding of cancer, fear of cancer, suspicion of cancer as an infectious disease).

The results showed that the symptoms discrimination in male students was more obvious, which may be related to a poorer humanistic care empathy of male students compared with that of female students [43]. We also found that students with more access to cancer knowledge were more likely to hold cancer symptoms discrimination, which may indicate that more access to cancer information does not guarantee the accuracy of the information itself or the correct understanding of relevant knowledge in students. These students, full of vigor and vitality, are receiving general higher education or secondary vocational education. They are expected to be the core in the progress of the society after graduation, therefore their ideological and behavioral characteristics deserve our attention and reflection. The discrimination of cancer symptoms among the university/college students reflects the problem in China’s school health education to some extent. Most of the universities/colleges and secondary vocational schools in China have set up health education-related courses, but those courses are often marginalized. There is a lack of health education teaching management, a lack of teaching hardware and software resources. Also, the teaching systems and teaching plans are unsound and poorly prepared, and the teaching contents are unsystematic and incomprehensive. All the factors contribute to the poor health awareness, poor health literacy and low disease awareness in the students. In addition, the lack of family health education, the wrong guidance of public opinion, feudal superstition and other factors may cause attitudes discrimination such as “Cancer is karma”, “Cancer is contagious”, “It is unlucky to associate with cancer patients”, and “Cancer patients are not clean” in the students [33]. This sort of attitude discrimination would further lead to behavioral discrimination. Intervention measures for these problems need to be further studied and discussed. The problem of cancer symptoms discrimination among medical college students could be a proof that the administrators in medical schools have not attached sufficient attention to medical humanities education. Some students are with insufficient medical humanistic empathy or even don’t possess any. Therefore, when cultivating medical students, professional skills and humanistic spirit should be given equal importance. And the integration of medical sciences and medical humanistic education is truly in need [44].

### Limitations

There are some limitations in this study. This study recruited the freshmen, sophomores and junior students only, because most of the seniors had started their internships outside the school and the inclusion of the seniors could lead to a low follow-up rate. The purpose of this study is to make a preliminary exploration of the cancer discrimination problems among university/college and secondary vocational school students, so strict probabilistic sampling wasn’t used. Due to the limitation of survey conditions, the test-retest reliability evaluation was not conducted in this study. At the time of this study, there was no authoritative Chinese version of a scale to measure cancer stigma to serve as a reference to conduct a correlation test for criterion validity. A recently published translation and validation of a Chinese version of the Cancer Stigma Scale might serve this purpose in future research [45]. As this study is a preliminary exploratory study, only 23 sociological, demographic and psychological variables that may affect the level of cancer symptoms discrimination are analyzed in this study, and new variables need to be included in subsequent studies. In addition, the geographical distribution of the investigation sites in this study is also relatively limited. The investigation was only carried out in some schools in central Yunnan province. In the future, the sample would be expanded to more areas in China including people with various social and demographic characteristics.

## Conclusions

There are different degrees of cancer symptoms discrimination in the university/college and secondary vocational school students. This CSDS scale has a good reliability and validity in the students, and can be used to evaluate the risk of cancer symptoms discrimination among healthy students. It can be generalizable to other non-cancer groups and can provide more theoretical basis for the reduction and elimination of cancer discrimination when reevaluation of reliability and validity is accomplished. In this way, the social support of cancer patients can be strengthened more actively, the quality of life of patients can be improved, and the survival time of patients can be prolonged.

If the scale is of help to a researcher, s/he has our permission to translate it into other languages, and to apply it to cancer-related studies, on the premise that this literature is cited and commercial interests are not involved.

## Supplementary information

**Additional file 1.** CSDS Semi-structured Interview Guide.

## Data Availability

The data used and analyzed during the current study are available from the corresponding author on reasonable request.

## Abbreviations

CSDS: Cancer symptoms Discrimination Scale
CR: Composite reliability
GFI: Goodness of fit index
CFI: Comparative fit index
AGFI: Adjusted goodness of fit index
NFI: Normed fit Index
RMSEA: Root mean squared error of approximation
TLI: Tucker-Lewis index
NFI: Normed fit index
CFA: Confirmatory factor analysis
KMO: Kaiser-Meyer-Olkin
I-CVI: Item-level content validity index

## References

[1] Kexin S, Rongshou Z, Siwei Z (2019). Report of Cancer Incidence and Mortality in Different Areas of China, 2015. China Cancer.

[2] Jim HS, Pustejovsky JE, Park CL (2015). Religion, spirituality, and physical health in cancer patients: A meta-analysis. Cancer.

[3] Yi J, Kim MA, Tian T (2014). Perceived long-term and physical health problems after cancer: adolescent and young adult survivors of childhood cancer in Korea. Eur J Oncol Nurs.

[4] Saquib N, Pierce JP, Saquib J (2011). Poor physical health predicts time to additional breast cancer events and mortality in breast cancer survivors. Psychooncology.

[5] Fisher P (2016). Cancer and quality of life. Homeopathy.

[6] Kasvis P, Vigano M, Vigano A (2019). Health-related quality of life across cancer cachexia stages. Ann Palliat Med.

[7] Williams GR, Pisu M, Rocque GB (2019). Unmet social support needs among older adults with cancer. Cancer.

[8] Iannarino NT, Scott AM, Shaunfield SL (2017). Normative Social Support in Young Adult Cancer Survivors. Qual Health Res.

[9] Breuer N, Sender A, Daneck L (2017). How do young adults with cancer perceive social support? A qualitative study. J Psychosoc Oncol.

[10] Fitzmaurice C, Allen C, Global Burden of Disease Cancer Collaboration (2017). Global, Regional, and National Cancer Incidence, Mortality, Years of Life Lost, Years Lived With Disability, and Disability-Adjusted Life-years for 32 Cancer Groups, 1990 to 2015: A Systematic Analysis for the Global Burden of Disease Study. JAMA Oncol.

[11] Robb K, Stubbings S, Ramirez A (2009). Public awareness of cancer in Britain: a population-based survey of adults. Br J Cancer.

[12] Kuzgunbay B, Yaycioglu O, Soyupak B (2013). Public awareness of testicular cancer and self-examination in Turkey: a multicenter study of Turkish Urooncology Society. Urol Oncol.

[13] Ettridge KA, Bowden JA, Chambers SK (2018). “Prostate cancer is far more hidden…”: Perceptions of stigma, social isolation and help-seeking among men with prostate cancer. Eur J Cancer Care (Engl).

[14] Fleisch Marcus A, Illescas AH, Hohl BC (2017). Relationships between social isolation, neighborhood poverty, and cancer mortality in a population-based study of US adults. PLoS One.

[15] Ernst J, Mehnert A, Dietz A (2017). Perceived stigmatization and its impact on quality of life - results from a large register-based study including breast, colon, prostate and lung cancer patients. BMC Cancer.

[16] Chen W, Zheng R, Baade PD (2016). Cancer statistics in China, 2015. Ca Cancer J Clin.

[17] Vermeulen R, Downward GS, Zhang J (2019). Constituents of Household Air Pollution and Risk of Lung Cancer among Never-Smoking Women in Xuanwei and Fuyuan. China. Environ Health Perspect.

[18] Downward GS, Hu W, Large D (2014). Heterogeneity in coal composition and implications for lung cancer risk in Xuanwei and Fuyuan counties, China. Environ Int.

[19] Li B, Ruan Y, Ma L (2015). Pathogenesis sequences in Gejiu miners with lung cancer: an introduction. Front Med.

[20] Marahatta SB, Adhikari B, Mishra SR (2015). Association of Previous Smoking Habit and Perceived Social Discrimination with the Risk of Multi-Drug Resistant Tuberculosis in Central Nepal. J Nepal Health Res Counc.

[21] Su X, Lau JT, Mak WW (2013). Perceived discrimination, social support, and perceived stress among people living with HIV/AIDS in China. AIDS Care.

[22] Le TV, Vu TTM, Mai HT (2019). Social Determinants of Stigma and Discrimination in Vietnamese Patients with Chronic Hepatitis B. Int J Environ Res Public Health.

[23] Wickham S, Taylor P, Shevlin M (2014). The impact of social deprivation on paranoia, hallucinations, mania and depression: the role of discrimination social support, stress and trust. PLoS One.

[24] Li J, Huang YG, Ran MS (2018). Community-based comprehensive intervention for people with schizophrenia in Guangzhou, China: Effects on clinical symptoms, social functioning, internalized stigma and discrimination. Asian J Psychiatr.

[25] Lasalvia A, Zoppei S, Van Bortel T (2015). Global pattern of experienced and anticipated discrimination reported by people with major depressive disorder: a cross-sectional survey. Lancet.

[26] Loveday M, Hlangu S, Furin J (2019). Healthcare Provider Discrimination toward Pregnant Women with Rifampin-Resistant Tuberculosis. Emerg Infect Dis.

[27] Jialiang H (2005). On the socio-psychological causes of social discrimination and possible solutions: An analysis of social discriminations from the perspective of socio-psychology. Si Xiang Zhan Xian.

[28] Shepherd MA, Gerend MA (2013). The blame game: cervical cancer, knowledge of its link to human papillomavirus and stigma. Psychol Health.

[29] Cataldo JK, Brodsky JL (2013). Lung Cancer Stigma, Anxiety, Depression and symptoms Severity. Oncology.

[30] Mosher CE, Sharon DB (2007). Death anxiety and cancer-related stigma: a terror management analysis. Death Stud.

[31] Martin ML, Halling K, Eek D, et al. “Lower abdominal pains, as if I was being squeezed…in a clamp”: A Qualitative Analysis of Symptoms, Patient-Perceived Side Effects and Impacts of Ovarian Cancer. Patient. 2020;13(2):189–200. 10.1007/s40271-019-00393-8 PMC7075817 31691205

[32] Kissane DW, Patel SG, Baser RE (2013). Preliminary evaluation of the reliability and validity of the Shame and Stigma Scale in head and neck cancer. Head Neck.

[33] Feng LS, Li XY, Wang HR (2018). Development and validation of the cancer self-perceived discrimination scale for Chinese cancer patients. Health Qual Life Outcomes.

[34] Feng LS, Wang YF (2016). Causes and influences of perceived discrimination in patients with cancer. Medicine Philosophy.

[35] Bubis LD, Delibasic V, Davis LE, et al. Patient-reported symptoms in metastatic gastric cancer patients in the last 6 months of life. Support Care Cancer. 2020; 10.1007/s00520-020-05501-1. 10.1007/s00520-020-05501-1 32415385

[36] Kim JHJ, Bright EE, Williamson TJ, et al. Transitions in coping profiles after breast cancer diagnosis: implications for depressive and physical symptoms. J Behav Med. 2020. 10.1007/s10865-020-00159-w. 10.1007/s10865-020-00159-w PMC7736058 32535673

[37] Bao Y, Pan S (2015). Differences-seeking Approach in Qualitative Method and Its Theoretical Foundation. Sociological Review of China.

[38] Zhonglin W, Kittai H, Marsh HW (2004). Structural equation model testing: cutoff criteria for goodness of fit indices and chi-square test. Acta Psychologica Sinica.

[39] Cheng S, Hu Y, Pfaff H, et al. The Patient Safety Culture Scale for Chinese Primary Health Care Institutions: Development, Validity and Reliability. J Patient Saf. 2020. 10.1097/PTS.0000000000000733. 10.1097/PTS.0000000000000733 PMC7908859 32404850

[40] Clark LA, Watson D (1995). Constructing validity: Basic issues in objective scale development. Psychol Assess.

[41] Hendriks JM, Crijns HJ, Tieleman RG (2013). The atrial fibrillation knowledge scale: Development, validation and results. Int J Cardiol.

[42] Labroca P, Chiesa G, Laroyenne I (2019). Quality of life assessment following amputation for septic shock: a long-term descriptive survey after symmetric peripheral gangrene. J Crit Care.

[43] Shen Z, Yang X, Han B (2013). Development and application of scale of human caring capacity for medical students. J Third Mil Med Univ.

[44] Song P, Tang W (2017). Emphasizing humanities in medical education: Promoting the integration of medical scientific spirit and medical humanistic spirit. Biosci Trends.

[45] Ye X, Liu HY, Lu SR (2019). Translation and validation of the Chinese version of the Cancer Stigma Scale. J Oncol Pharm Pract.

