# Supplementary material for: Development and validation of the cancer symptoms discrimination scale: a cross-sectional survey of students in Yunnan, China
Source: BMC Palliat Care. 2020 Oct 12;19:156. doi: 10.1186/s12904-020-00662-6 (PMC7552442; doi:10.1186/s12904-020-00662-6)
Supplement: Supplementary file 1 — Additional file 1. CSDS Semi-structured Interview Guide. [file 12904_2020_662_MOESM1_ESM.docx]

**Additional File 1. CSDS Semi-structured Interview Guide**

Guiding questions

Do you agree to participate in the study?

I would like to appreciate your help in responding to this semi-structured interview. If you are comfortable, let we start the discussion with:

1) Have you ever socialized with cancer patients?

2) Have you ever discriminated against cancer patients?

3) How many cancer symptoms do you recognize?

4) Have you ever discriminated against cancer symptoms?

5) Which cancer symptoms did you discriminate against?

6) Why did you discriminate against these cancer symptoms?

Thank you for your time.
